# Improve Safety of Antipsychotic Prescribing in Dementia

**DOI:** 10.1192/bjo.2026.11445

**Published:** 2026-06-30

**Authors:** Niranchana Jeyakanthan, Erum Nomani

**Affiliations:** CNWL NHS Trust, Harrow, United Kingdom

## Abstract

**Aims:**

This Quality Improvement project aimed to ensure safer prescribing of antipsychotics in dementia by standardising information provision, enhancing clinician confidence, and promoting psychosocial interventions as first-line treatments.

**Methods:**

We conducted an audit to assess the frequency of discussions regarding antipsychotic use and information provision to patients, families and carers. A structured intervention was introduced, including:

–Development and distribution of an evidence-based information leaflet.

–Staff training sessions on antipsychotic prescribing in dementia.

–Behaviour that Challenges (BtC) consultations to help caregivers understand distress triggers and alternative management strategies.

**Results:**

A sustained improvement was achieved, with 100% of patients and caregivers receiving information leaflets for seven consecutive months. We introduced BtC consultations which led to improved understanding of behavioural triggers and better management. After these interventions we reassessed reliance on antipsychotic prescriptions and found an overall reduction in the number of prescriptions.

**Conclusion:**

This QI project successfully enhanced the safety of antipsychotic prescribing in dementia by improving information-sharing and prioritising non-pharmacological interventions. Future steps include expanding leaflet distribution across older adult services and continued collaboration with pharmacies and primary care teams.

